# Understanding the role of contrasting urban contexts in healthy aging: an international cohort study using wearable sensor devices (the CURHA study protocol)

**DOI:** 10.1186/s12877-016-0273-7

**Published:** 2016-05-05

**Authors:** Yan Kestens, Basile Chaix, Philippe Gerber, Michel Desprès, Lise Gauvin, Olivier Klein, Sylvain Klein, Bernhard Köppen, Sébastien Lord, Alexandre Naud, Hélène Payette, Lucie Richard, Pierre Rondier, Martine Shareck, Cédric Sueur, Benoit Thierry, Julie Vallée, Rania Wasfi

**Affiliations:** Montreal University Research Center (CRCHUM), 850, rue St-Denis, Montréal, QC H2X 0A9 Canada; École de Santé Publique de l’Université de Montréal (ESPUM), 7101, rue du Parc, Montréal, QC H3N 1X9 Canada; Inserm, UMR-S 1136, Pierre Louis Institute of Epidemiology and Public Health, Faculté de médecine Saint-Antoine, 27 rue Chaligny, cedex 12, Paris, 75571 France; Luxembourg Institute of Socio-Economic Research, 11, Porte des Sciences, Esch-sur-Alzette, L-4366 Luxembourg; École d’urbanisme et d’architecture de paysage, Université de Montréal, 2940, chemin de la Côte-Sainte-Catherine, Montréal, QC H3C 3 J7 Canada; Canada Research Center on Aging, CIUSSS de l’Estrie-CHUS, Faculty of Medicine & Health Sciences, University of Sherbrooke, Sherbrooke, QC Canada; Faculty of Nursing, Université de Montréal, Montreal, QC H3C 3 J7 Canada; London School of Hygiene and Tropical Medicine, London, UK; UMR Géographie-Cités, 13 rue du Four, Paris, 75006 France; Département d’Ecologie, Physiologie et Ethologie; Institut Pluridisciplinaire Hubert Curien, 23, rue Becquerel, Strasbourg, 67087 France

**Keywords:** Healthy aging, Daily mobility, Physical activity, Social participation, Social networks, Wearable sensors, GIS, Spatial epidemiology, Mixed methods

## Abstract

**Background:**

Given the challenges of aging populations, calls have been issued for more sustainable urban re-development and implementation of local solutions to address global environmental and healthy aging issues. However, few studies have considered older adults’ daily mobility to better understand how local built and social environments may contribute to healthy aging. Meanwhile, wearable sensors and interactive map-based applications offer novel means for gathering information on people’s mobility, levels of physical activity, or social network structure. Combining such data with classical questionnaires on well-being, physical activity, perceived environments and qualitative assessment of experience of places opens new opportunities to assess the complex interplay between individuals and environments. In line with current gaps and novel analytical capabilities, this research proposes an international research agenda to collect and analyse detailed data on daily mobility, social networks and health outcomes among older adults using interactive web-based questionnaires and wearable sensors.

**Methods/Design:**

Our study resorts to a battery of innovative data collection methods including use of a novel multisensor device for collection of location and physical activity, interactive map-based questionnaires on regular destinations and social networks, and qualitative assessment of experience of places. This rich data will allow advanced quantitative and qualitative analyses in the aim to disentangle the complex people-environment interactions linking urban local contexts to healthy aging, with a focus on active living, social networks and participation, and well-being.

**Discussion:**

This project will generate evidence about what characteristics of urban environments relate to active mobility, social participation, and well-being, three important dimensions of healthy aging. It also sets the basis for an international research agenda on built environment and healthy aging based on a shared and comprehensive data collection protocol.

## Background

Although some components of healthy aging have been linked to various dimensions of the built environment [1], a detailed understanding of the processes linking environments to aging is still lacking. Given the challenges of aging populations and growing urbanization in industrialised nations [2–4], calls have been issued for more sustainable urban (re)-development and implementation of local solutions to address global environmental and health issues [5, 6]. The ways our cities are designed represent both a challenge as well as an opportunity for modifying and improving living conditions, environment, and health. How environments influence health is particularly relevant among older adults, a population segment that may be more place-bound than their younger counterparts [7–9]. As a consequence, the influence of proximal environmental living conditions may increase as people age.

Along this line, the model of neighbourhood effects on aging of Glass and Balfour [10] provides an appropriate conceptual underpinning to explore relations between urban environments and healthy aging. Drawing from Lawton’s ecological model of aging [11, 12], the degree of person–environment fit determines the degree of adaptation and subsequent health. Extending Lawton’s proposition and integrating the notions of positive and negative effects, Glass and Balfour hypothesise that neighbourhood environments can contribute to healthy aging either in salutogenic or deleterious ways. The neighbourhood can either impose demands or barriers on the individual - environmental press-, or it can facilitate adaptation - environmental buoy. Socio-environmental conditions, social integration, physical aspects of places, and services/resources are part of these environmental presses or buoys, which in turn interact with personal competencies to produce differential person–environment fits. In line with the notion of environmental buoys and pressers, an environment rich in services and amenities, with appropriate urban forms or land use patterns may favour active living or social participation, and thereby positively contribute to sustainability and healthy aging. Conversely, an environment that is scarce in opportunities may become unwieldy for seniors and contribute to unhealthy aging. Such opportunities or barriers can be objectively assessed using GIS-derived indicators, and be combined to GPS logging for objective evaluation of exposures in activity spaces [13, 14]. They can also be evaluated as perceived or experienced elements of everyday interactions with one’s surroundings.

Until now, the operational testing of such hypotheses, − including studies on walking or physical activity [7, 15–17], diet [18], or social participation among seniors [7, 19, 20] – have mainly consisted in evaluating potential opportunities in the residential environment, based on administrative units, or through ego-centred proximity or density measures. Built environments [21–25], social networks [26–28], and mobility [29–31] are increasingly the focus of attention, both because of their potential role in contributing to healthy aging, and because these dimensions may be amenable for intervention [32–37]. Characteristics of social networks [26, 38–40], and closely related notions of support perceived or received from others [36, 41–43], level of social integration [36, 44–46], and a sense of isolation and loneliness [29, 47, 48] have also been both theoretically and empirically identified as predictors of health status, active living and well-being. However, few studies have yet proposed a comprehensive protocol addressing the complex interplay between detailed individual social network, cultural or financial resources, the presence of local environmental resources or hazards, transportation infrastructures, urban form, and aging-related health outcomes, while accounting for daily mobility.

Calls have however been made to overcome the ‘residential’ or ‘local’ trap [49, 50] – that is, the potential limitation of considering only administratively-defined residential areas as opposed to broader activity space-based measures of people-place interactions accounting for daily travel patterns [51]. Self-reported questionnaires have been used to assess older adults’ out-of-home destinations or activity places. As an example, the life-space questionnaire has been used to assess non-residential activity spaces or destinations among older adults [52]. Such measures of daily travel patterns have been associated among older adults with frailty [9, 53], use of power mobility devices [54], eye-disease [55], with post-hospitalization [56] or geriatric rehabilitation [57] in relation to quality of life [58], and such associations have been evaluated in various cultural contexts [8]. Although insightful, such life-space questionnaires do not collect precise, fine-grained and objective information on activity destinations, daily mobility and related physical activity patterns. In short, they are not adapted if precise collection of spatial information is needed. Allowing respondents to search and locate destinations on a map can bridge that gap, while novel ubiquitous wearable sensors further allow gathering continuous information on people’s location, levels of physical activity, perceptions, attitudes or feelings [59–63]. Combining wearable Global Positioning System (GPS) receivers and accelerometers allows obtaining precise information on activity locations and trips between destinations, and estimates of walking and energy expenditure in each of these segments over the daily schedules [64, 65]. Such objective assessments of mobility and physical activity can be used to further guide qualitative interviews, and support mixed method approaches combining objective and subjective evaluations of people-place interactions.

In a context of aging populations, calls have been made for sustainable (re)-development of our cities. Developing and applying novel tools and methods are needed to help explain how older adults interact with places and how such interactions favor healthy aging. Recent developments in wearable sensor platforms allowing a continuous assessment of daily mobility, physical activity, social participation, and perception of urban environments offer important research possibilities to better understand urban determinants of healthy aging. Such tools can be used to gather precise and unprecedented information on how older adults interact with places on a daily basis and on how these interactions translate into health profiles. Yet, to tackle such a complex issue, there is a need for multidisciplinary expertise and conduction of analyses in contrasted settings, to increase external validity and usefulness of research results. We have joined forces between three research teams in three countries, to co-develop and apply a common research protocol aimed at better understanding the interplay between urban environments, mobility and healthy aging.

## Methods

### Objectives and hypotheses

**Fig. 1:**
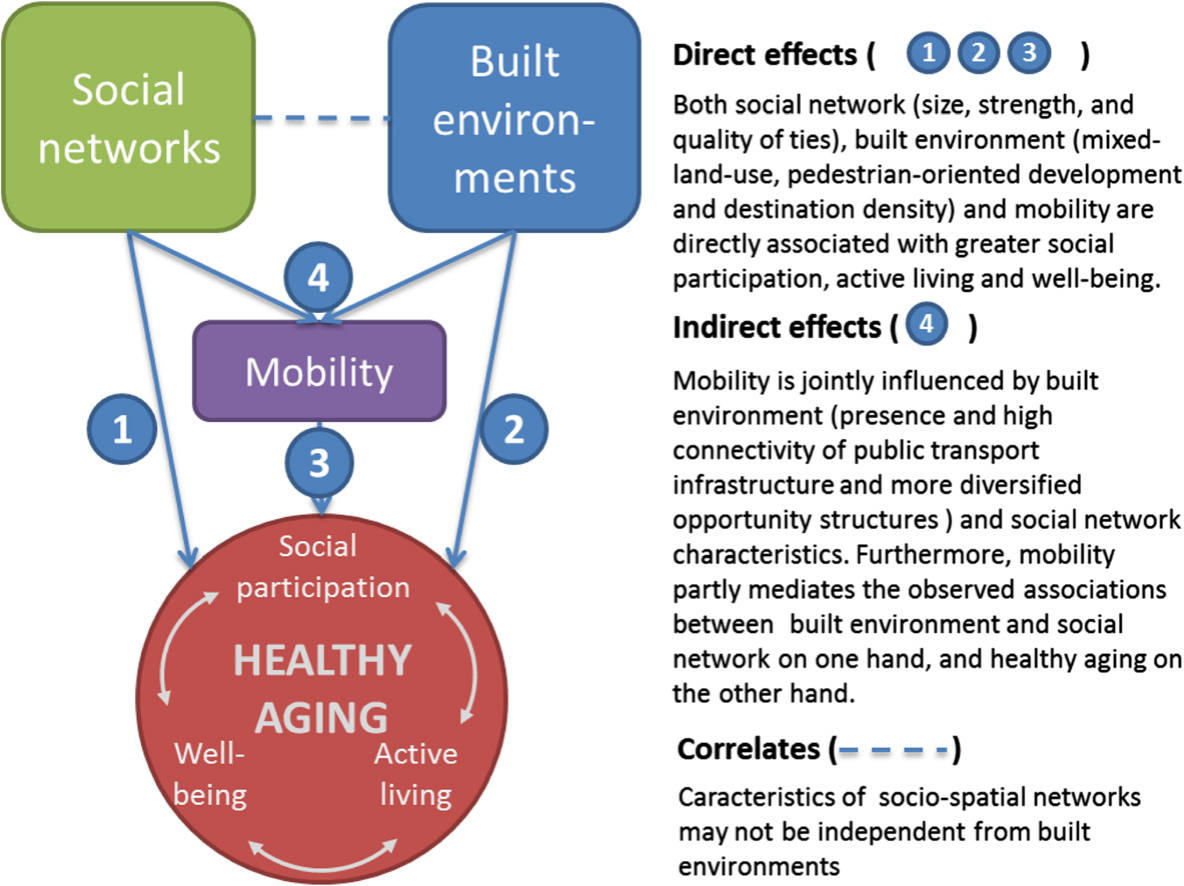
Main hypothesised pathways linking social networks, built environments, and mobility to healthy aging

The CURHA (Contrasted Urban settings for Healthy Aging) project develops an international platform and research agenda to collect and analyse extensive data on daily mobility, social networks, and healthy aging outcomes. The project includes two pre-existing and one developing cohorts of adults or older adults living in contrasted urban settings in Montreal, Paris, and Luxembourg. The combination of data being collected – both of quantitative and qualitative nature-, existing GISs, and advanced analytical methods will provide new opportunities to disentangle the people-environment interactions linking urban contexts to healthy aging. Particularly, this project will provide evidence about how characteristics of urban environments and social networks relate to active living, social participation, and well-being, while accounting for daily mobility. Resulting evidence will help guide interventions to improve urban contexts promoting healthy aging. Figure 1 presents hypotheses to be explored, including direct and indirect effects.

### Description of participants/cohorts

This project builds on two existing cohorts, − the NuAge cohort in Canada, and the RECORD cohort in France-, and further launches a new cohort of older adults in Luxemburg.

Canada: A sub-sample of 175 participants living in urban and suburban neighbourhoods of Montreal and Sherbrooke in Quebec Province will be recruited from The Quebec Longitudinal Study on Nutrition and Successful Aging (NuAge) cohort, launched in 2003. NuAge is a prospective study that included 1793 cognitively intact and functionally independent elders aged 67–84 years upon recruitment [66]. The cohort was drawn from an age- and sex-stratified random sample from the Quebec Medicare database (RAMQ – Régie de l’assurance-maladie du Québec) for the regions of Montreal, Laval, and Sherbrooke in the province of Québec, Canada. All NuAge participants were tested annually between 2003 and 2007 using a series of nutritional, functional, medical, biological, and social measurements. In 2007, a satellite study was launched to evaluate the relation between residential built environment characteristics and healthy aging among Montreal participants of the NuAge Cohort. NuAge participants residential locations were linked to environmental predictors using MEGAPHONE, a comprehensive GIS providing detailed local built and social environments indicators hypothesised to be associated with healthy aging outcomes. Analyses have revealed associations between accessibility to resources and walking [67], depression [68], social participation [69], and diet [70].

France: In France, no additional funding was obtained for this CURHA project, but a number of analyses will be possible with participants of the RECORD GPS Study. The RECORD Cohort is a cohort of 7,290 adults and older adults recruited between March 2007 and February 2008 for the purpose of analysing the influence of geographic life environments on a series of health behaviours and health outcomes [71]. Recruitment was done among people visiting their preventive clinical examination centre (Centre d’Investigation Préventive et Clinique), which is covered by the health insurance of working, unemployed, and retired salaried workers. Participants, aged 30 to 79 at baseline, were drawn from 111 municipalities from the Paris region and from 10 boroughs of the city of Paris. All participants have completed a series of questionnaires covering socio-demographic dimensions, health behaviour, psychosocial dimensions, various dimensions relating to their perception of places, and medical history. Complementary physiological and biological data was further obtained through a 2-h medical examination, providing objective measures of height, weight, waist circumference, blood pressure, and lipid and glycemic profiles. The second wave, started in 2011, is ongoing, with additional participants recruited to compensate for loss to follow-up and to increase sample size. Early 2012, in collaboration with the Spherelab group, the GPS RECORD Study was launched. A sub-sample of 234 participants wore a GPS and accelerometer device for a one week period, and answered prompted recall questionnaires for further collection of qualitative information on the nature of activities, modal choice, and attachment to places, and for validation of automatic detection of activity places and trips [72], and modes of transportation. In 2015, a novel project was funded by Agence Nationale de la Recherche to further extend data collection among a subsample of 400 older adults within RECORD, allowing collection of social network measures as partly done in the CURHA project. With this new project, comparability with the Canadian and Luxembourg cohorts set up in CURHA will increase. Also of interest for future collaboration is the MINDMAP project funded by the European Research Council, which aims to investigate the bidirectional processes linking built environments and mental health, i.e., both how the depressive symptomatology influences the perception of, navigation in, and use of one’s environment, and how features of visited built environments imply daily variations in depressive symptomatology. In this protocol, older RECORD participants, carrying GPS receivers and accelerometers, will answer short surveys prompted on a smartphone, and will undergo an assessment galvanic skin response (electrodermal activity as a potential marker of stress) using the SenseWear device.

Luxembourg: In Luxembourg, a new cohort is being developed for this project by the LISER Group. A target recruitment of 500 participants aged 65 and up is based on a sampling scheme using Social Security administrative files. These anonymized micro-data allow the selection of all elderly while accounting for some socio-demographic criteria (age and gender) and their place of residence characteristics. The final sample will include 100 participants within each of the five following types of urban environments: core city of Luxembourg, city of Esch-sur-Alzette, sub-center cities in the south of the Grand Duchy, primary suburban area with high level of amenities, and secondary suburban area with low level of amenities.

The Luxembourg cohort will furthermore include social background, migration history, educational and employment history, social and professional status, class membership, housing and dwelling characteristics, respondent’s autonomy and mobility limitations and individual strategies to overcome these limits. A chapter on social and medical care, accessibility, and availability of public and private care providers will also cover information about the needs for support and assistance.

#### Inclusion and exclusion criteria

Inclusion criteria require participants to be aged 65 or up, to present limited or no cognitive impairment, and to not have moved in a long-term care facility or outside the study areas. In Montreal, members of the NuAge cohort from which participants are drawn are eligible if they get a score of 17 or higher on a scale of 26 to the telephone version of the Mini-Mental state examination.

### Data collection and measures

In order to optimise pooled and comparative analyses, the data collection and analytic procedures builds on shared protocols. One focus of this project concerns the detailed assessment of daily mobility and participants’ social networks, in order to assess their relation to environmental contexts and healthy aging, including active living, social participation and well-being. Daily mobility is measured both through self-report instruments using VERITAS, an interactive map-based questionnaire [73] - to which a social network component was added-, and objectively using wearable sensors including GPS tracking and an accelerometer. Finally, qualitative assessment of place experiences further tries to shed light on daily mobility means and meanings.

**Fig. 2:**
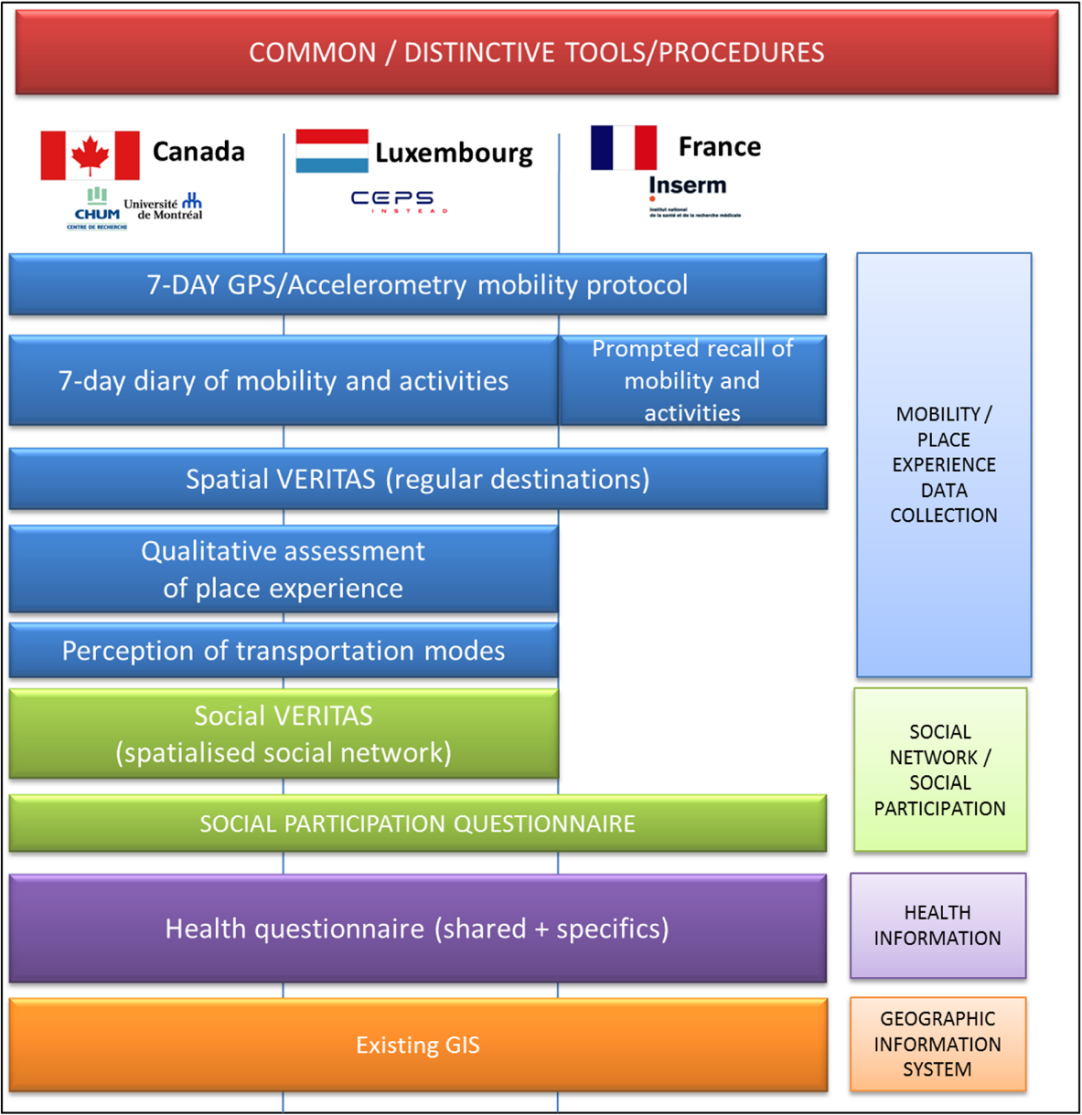
Depiction of common / distinctive tools and procedures of CURHA protocol across three countries

However, because this proposal builds partly on existing cohorts, certain validated questionnaires on health behaviours, health outcomes and individual-level determinants have already been defined in certain settings. In order to limit the additional burden imposed on the participants, we only added novel questionnaires when strictly necessary, to maximize comparability of settings. Table 1 and Fig. 2 illustrates common tools/procedures of the CURHA protocol, and Table 1 provides some examples of variables of interest.

**Table 1:** Protocol components, measures and variables of interest

Protocol component	Data/measures	Derived variables of interest (non-exhaustive list)
7-day GPS and accelerometer	Daily mobility, physical activity, sedentary time	Activity space indicators, time spent in various locations, distance travelled, transportation modes used, number of steps, sedentary time, light activity, mvpa
7-day diary of self-reported destinations and social contacts	Complementary information on transportation modes used, destination types, and social contacts	Transportation modes, number of in-person social contacts
VERITAS socio-spatial questionnaire	Location of regular destinations, frequency of visit, social network characteristics, novel socio-spatial indicators	Regular destination activity space indicators, social network size, strengths, spatial print of social network
Qualitative assessment of mobility and place experience (Go-along method)	Qualitative assessment of sens of place, meanings of mobility	Understanding meaning of mobility, urban environment characteristics, and social networks for aging
CAPI Questionnaires	Numerous outcomes and control variables including health and perceived environment	Health outcomes of interest include well-being, happiness, mental health, physical activity, social participation
Geographic Information Systems	Land use, transportation infrastructure, ressources, greenness, neighbourhood composition	Land use mix, accessibility to local resources including transportation, libraries, community organisations, social composition indicators

#### Administered questionnaires

##### Health and neighbourhoods

Computer-assisted personal interviewing (CAPI) will be performed and will include the administration of questionnaires on health (Mini-mental State Examination (MMSE), Geriatric Depression Scale (GDS), Physical Activity Scale for Elderly (PASE), Subjective Happiness Scale, Short Form (36) Health Survey), on neighbourhoods (Perceived accessibility to resources, social capital), and on mobility (Life Space Questionnaire, perceptions regarding various transportation modes).

### VERITAS*-social*: collecting regular activity places and social networks

**Fig. 3:**
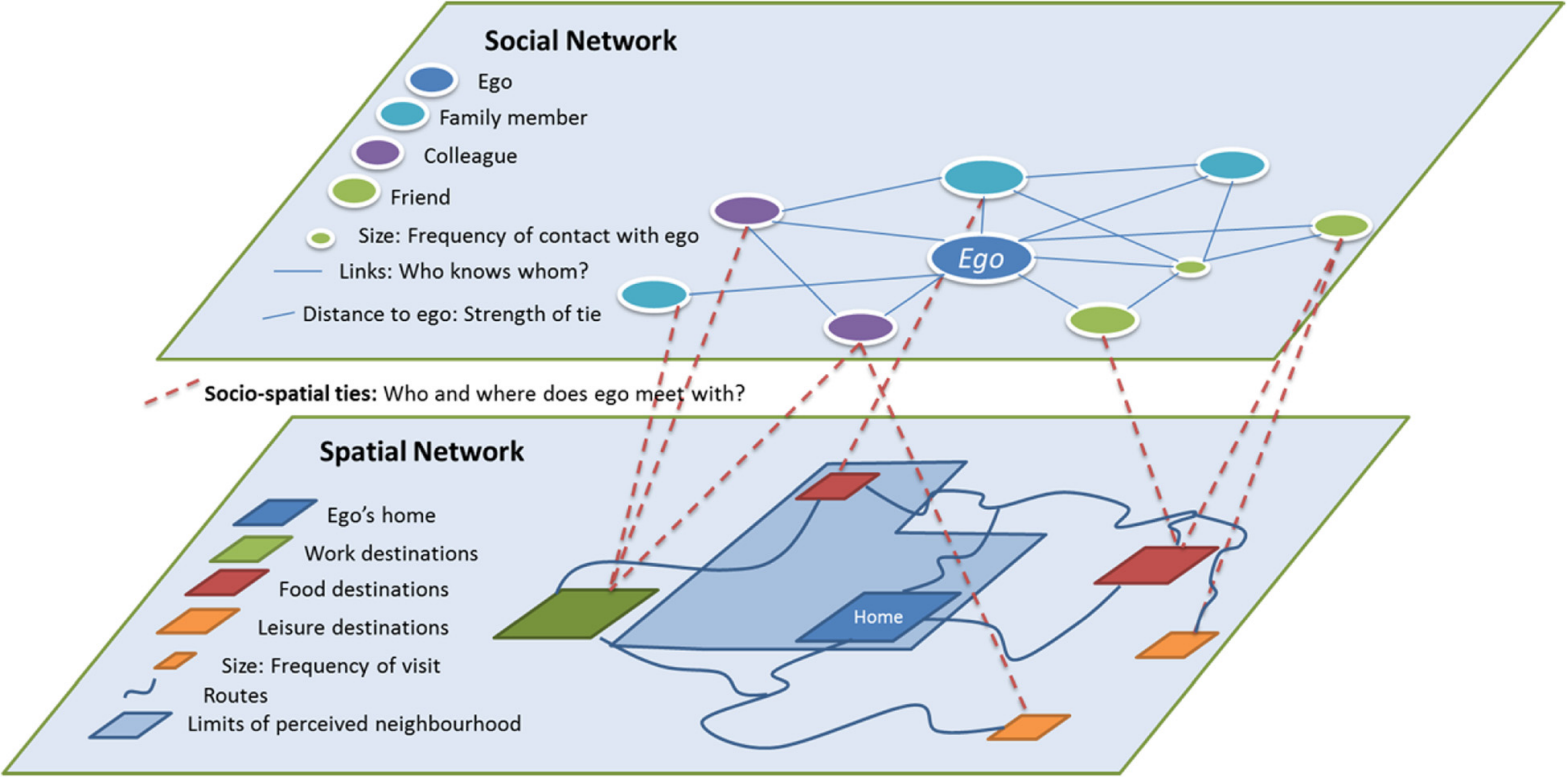
Social and spatial network components collected with VERITAS-Social questionnaire or GPS with diary/prompted recall

We will administer VERITAS, an interactive map-based questionnaire designed to collect information on daily mobility, including identification of regular destinations and frequency of visit, perceptions of neighbourhood limits, and complementary data describing activity locations, routes, or transportation modes. The questionnaire presents questions about ‘where’ a number of activities are being conducted. Using map search capabilities, the destinations are found on the map and confirmed by the participant. Consequently, geographic coordinates, name of destination, and complementary information including frequency of visit or mode of transportation used to get there are saved in a spatial database. To this existing framework, we will add a social network module. When respondents will document a given destination, they will also be asked to provide information on contacts from one’s network with whom they usually go there. Inspired from the use of name generators [74, 75], this procedure allows documentation of the ego-centered social - and spatialized - network. Network members are described in terms of age, gender, type of connection (husband/wife, other relative, friend, colleague, acquaintance), and if they live in the same neighbourhood or same city as the participant. At the end of the questionnaire, participants are presented with all the network members they have identified throughout the spatial questionnaire. They are further required to mention other network members that may not have mentioned yet (i.e. with whom they don’t share a common activity/destination) and are then asked to document for all members A) ‘with whom they discuss important matters’, B) ‘with whom they like to socialize’, as in regular social network questionnaires [76, 77], as well as C) who in their network knows whom. These questions allow assessing the emotional closeness between participants and alters, and identification of relationships between network members. Figure 3 illustrates both social and spatial network information collected through VERITAS-Social. Within the HANC protocol, VERITAS will be used to collect social network data from the list of places visited and additional spatial locations visited from the list of social network contacts.

#### GPS and accelerometry mobility protocol

We will collect continuous 7-day information on mobility and physical activity. To do so, we will equip participants with sensors offering the capacity to collect GPS tracks and raw accelerometry. In Montreal and Luxembourg, we will use the SenseDoc 2.0, a wearable multisensory device originally developed by the Spherelab group, and now marketed by Mobysens Technologies Inc. The SenseDoc is a compact multisensor device with high-sensitivity GPS, a tri-axial accelerometer (ADXL 345), short-range RFID (ANT+ and OpenBeacon) capacities for addition of complementary sensors, and cellphone capabilities for possible real-time transmission of data. The device has been specifically engineered to suit varied populations including children, patients following rehabilitation programs, and older adults, with minimal requirements for manipulation, no screen, and an extended battery life requiring minimal recharge. For this study, we will only use the GPS and accelerometer functions. For the RECORD GPS Study, an Actigraph GT3X and a QStarz BTX1000 GPS tracker were used.

A number of data processing methods and algorithms have been developed in collaboration between Kestens’ & Chaix’ teams, specifically through work on the RECORD GPS Study, and will be applied in this study [78, 79].

In order to validate the derived information on mobility and to collect detailed social contact information during the 7-day period, we will ask participants to fill in a travel diary where they will report information on mobility and social contacts (start and end times of a trip, location of activities, with whom they did the activity). In Paris, the RECORD GPS Study uses both a travel diary and a prompted recall survey at the end of the 7-day period, to validate GPS-detected activity locations, trips and estimated transportation modes, and identify conducted activities.

#### Quality assessment of place experience

Empirically the mixed qualitative-quantitative analysis of aging person’s mobility intends at three specific objectives: 1) understand in both complex and comparative perspectives (level of mobility, residential settings, and communities/cultures) how in a context of aging individuals adapt their mobility uses and their home territories (uses/representations); 2) reveal specific experiences and meanings of mobility/urban environments in connection with health, social participation, well-being, and environmental behaviors (following section) as well as regarding to elders’ representations of the city and of aging; 3) highlight individual motivations behind mobility choices and adaptation strategies in connection to specific urban sectors perceived (or not) as “practicable” environments. These specific empirical objectives are firstly analysed within the Canadian and Luxembourg national fieldworks and secondly developed as national outputs for cross-comparison between countries.

The qualitative section of the research will be closely linked to both GPS tracking and quantitative surveys. As part of a quantitative-qualitative mixed-approach, the qualitative section will be integrated and conducted concurrently with the objective evaluation of daily mobility. This will facilitate the linkage between qualitative databases and quantitative assessment of elders’ mobility and their environments. This qualitative section will also evaluate the acceptability of the methods, tools, and technologies proposed here.

Subsamples of roughly 20 participants in each research setting will be used for qualitative assessments. The exact final number of respondents required will be determined during the process, in accordance with the concept of saturation [80]. Semi-structured questionnaires with open-ended questions will firstly be conducted to obtain comprehensive images of individual experiences and meanings of activity spaces, mobility uses, and urban contexts in relation to the targeted outcomes of walking, social participation, and well-being. The qualitative perspective requires analyzing the discourse of elderly about their daily mobility, their experiences and meanings of home territories as aging persons [81]. Respondents will be invited to talk freely about their daily trips and about others relevant topics in relation to the quantitative surveys: usual outings and socio-spatial habits, openness to different modes of transportation, and the meanings of getting around in the city.

Go-along interviews will secondly be conducted in the neighbourhood of the participants. A typical activity (walking, shopping, etc.) from the respondent’s VERITAS questionnaire will be proposed in order to select a usual destination. The go-along interview is an accompanied trip where comments and observations can be noted directly in the experienced environment [82, 83]. The main advantage, in comparison or in addition to conventional interviews, is the direct immersion into the interviewee’s environment. The participant will choose the destination, the route and the mode of transportation. Both the qualitative questionnaire and the go-along interview will be saved in audio file to be transcribed, coded and analyzed. The go-along interview will be filmed and mapped for integration into a GIS, and analyzed in connection with quantitative data.

Outcomes of this mixed-method approach will be threefold: (i) establish the meaning of mobility in terms of spatial uses and daily routines, and highlight cultural differences in perceptions of mobility/behaviors between three national fieldworks, and (ii) uncover the complex relations between subjective experiences in person-environment relationships and observed (GPS and qualitative surveys) ageing, mobility, and health behaviors, and (iii) provide guidance for interpretation of quantitative results, including for non-significant but theoretically expected associations or even for eventually counter-intuitive findings.

The qualitative analysis software QDA Miner© will be used to code the interview transcriptions. The Miles and Huberman (1984) qualitative analysis principles will be applied on qualitative data in connection with GPS tracking and/or quantitative surveys outputs (e.g. mobility typology, patterns of social participation, etc.).

### Measures of interest

#### Mobility assessment

Raw 1-s GPS data obtained from the SenseDoc will be processed to derive detailed schedules of *activity locations* visited, *trips*, and estimations of *transportation modes* [84].

#### Physical activity

Physical activity evaluation will focus on the following outcomes: walking, sedentary behaviour, physical activity and active transportation. *Utilitarian and leisure walking* will be assessed using the Physical Activity Scale for the Elderly (PASE). The PASE is a brief questionnaire that requires participants to estimate the frequency and duration of a variety of physical activities and has been shown to have good reliability and validity among seniors [85, 86]. *Sedentary behaviour* will be assessed using the sedentary behaviour questionnaire for older adults developed in the context of the ‘Stand Up For Your Health’ study and validated with concurrent accelerometer measurement [87]. From the PASE and IPAQ questionnaires, we will retain the *monthly duration as well as frequency of utilitarian walking and leisure walking*. Finally, objective measures of *walking, sedentary activity, light, moderate and vigourous physical activity and number of steps* will be obtained from the 7-day accelerometer measurement period, with post-processed validation of transportation modes through linkage with diary information.

#### Social participation

Social participation will be evaluated using a 10-item scale adapted from the social portion of the ‘Elderly Activity Inventory Questionnaire’ [88] and Statistics Canada’s Participation and Activity Limitation Survey. The instrument assesses the involvement of respondents in activities such as attending cultural events, taking lessons, or volunteering. The social participation measure from the EAIQ will be the *frequency of engaging in a social activity* (nb of times per month) as well as from the VERITAS questionnaire, where we will obtain the *frequency of social activities conducted out-of-home* and further distinguish *activities conducted alone, or with a contact, either family member, friend, acquaintance or colleague*. The social activities can also be weighted in relation to location, such as by distance to home or if within or outside of the perceived limits of the neighbourhood.

#### Socio-spatial network

Information on social networks collected through VERITAS will allow derivation of a number of social network metrics. Traditional measures will include *network size* - the number of network members of a participant-, *network density* - the extent to which network members are connected to each other-, *boundedness* - the degree to which network members are defined and linked to the participant on the basis of traditional group structures (kin, work, friendship, neighbourhood), betweenness - how a participant is a bridge for other relationships-, homogeneity - similarity of characteristics of network members-, *homophily* – clustering of individuals with same characteristics. Other measures which are more related to the ties linking network members will include *strength of a link* as measured through the frequency and/or to the duration of contacts between two individuals, and the *nature of the link* such as in-person contact, phone contact or mail contact.

Furthermore, with the spatial information of network members’ places of residence and location of face-to-face contacts, a series of *socio-spatial measures* will be derived; either based on traditional spatial indicators or traditional social network indicators. For example, nodes in such a socio-spatial network can be either people or places, and ties can be between people, between places, or between people and places (See Fig. 3). Nodes of the socio-spatial network can be places or people, and ties can link people, places, or people to places. Distances between nodes can be spatial or virtual, based on strengths of ties or frequency of visit. In short, with this comprehensive data collection protocol on activity spaces and spatialized ego-centered social networks, we will be able to both ‘put the social network into its spatial context’ and ‘put the spatial experience into its social context’.

#### Perception of neighbourhood/environment

A number of questions document one’s *perceptions of the residential neighbourhood* - air quality, incivilities, noise, accessibility to healthy aging resources, presence of amenities such as benches, etc. Perceived *limits of the neighbourhood* were drawn on the interactive map in VERITAS, as well as *areas that are being avoided* because of safety concerns. Finally, a number of *residential choice* questions inquire if walkability, density of services, or accessibility to parks were considered criteria when choosing the neighbourhood.

#### Complementary environmental predictors derived from GIS

In each setting, participant’s place of residence as well as reported activity locations and GPS data will be linked to existing GIS platforms that contain rich information on urban contexts. Whereas a preliminary assessment has allowed identifying a series of database that are common to all settings, further integration work will be conducted to optimize similarities between derived variables. Whereas some data will be strictly identical in nature (road networks for example), others will possibly require re-compilation for comparability purposes (e.g. land use categorization for calculation of walkability indexes).

### Statistical analyses

We will develop quantitative models integrating the collected data on daily mobility, social networks, physical activity and perception of places into geographic information systems to develop new indicators and space-time modeling of health and place relations in regards to healthy aging. Mobility indicators will include multi-place measures based on time-geography principles, activity space indicators such as convex hulls, networks of usual places measures, and evaluation of daily trips. All available locational information stemming from the VERITAS questionnaire or the GPS monitoring will be linked through GIS to the built environment, including opportunities and barriers for walking or social participation.

The outcomes of interest will be analyzed either continuously, in categories, and as binary variables. Team members have a strong background in epidemiological modelling, with various specific expertise covering multilevel modelling, spatial models, social network analysis and complementary advanced statistical techniques.

Main predictors: Environmental predictors include perceived measures of the neighbourhood, objective GIS-derived indicators of access and exposure to resources, urban form, socio-economic composition, and crime. Environmental characteristics will be linked to individuals using ego-centered network buffers as definitions of local areas. These areas will both be used to compute environmental measures for the place of residence (residential exposure), for non-residential activity locations (activity space exposure) and for the GPS tracks (continuous exposure). Social network measures of interest will include network size, diversity, density, and strength. Control variables: a series of individual-level variables resorting to socio-economic position, functional capacity, co-morbidity, coming from the more general health questionnaires will be used as control variables. Spatial indicators: spatial indicators resorting from the VERITAS questionnaire and from the GPS traces such as activity space size or distance travelled during the week will be used either as outcome variables or as possible modifiers of the environment - social network - health relationship. Spatial metrics will be based on previous work conducted on the analysis of activity spaces based on VERITAS data.

## Discussion

This protocol offers key data collection components to unravel the complex interactions between daily mobility, social networks, urban environments, and healthy aging. Relevant data collected includes information on older adults’ regular destinations, social network structure and in-person encounters, daily mobility, physical activity and sedentary behaviour, and qualitative assessment of mobility experiences, on top of more classical self-reported health and perception questionnaires. With such rich data, and contrasted urban settings in different countries, the expected findings are potentially of interest to knowledge users shaping the city and other groups working on the improvement of older adults’ health. This protocol – with slight variations accounting for local specificities – is currently applied in Canada, Luxembourg, and France. We welcome teams interested in these topics to apply parts or the whole protocol described here in other settings to further increase contrasts and thereby maximise the capacity for comparative studies. Whereas it remains important to adapt to local contexts or specific research questions, we feel that the comprehensiveness of the procedures proposed here should fit a relatively large range of needs and settings. We welcome any suggestion from decision makers and knowledge users for complementary analyses that could further shed light on age-friendly urban environments.

### Ethics approval and consent to participate

This research was approved in Canada by the Comité d’Éthique de la Recherche of the Centre de Recherche du Centre Hospitalier de l’Université de Montréal (CRCHUM, #13.073), and in Luxembourg by the LISER ethical committee. All participants signed an informed consent form prior to participation.

### Consent for publication

Not applicable.

### Availability of data and material

The datasets generated through this study are deposited at the principal investigators’ laboratories. Please contact us if you are interested in conducting analyses.
