# Erratum to: Understanding the role of contrasting urban contexts in healthy aging: an international cohort study using wearable sensor devices (the CURHA study protocol)

**DOI:** 10.1186/s12877-016-0301-7

**Published:** 2016-07-05

**Authors:** Yan Kestens, Basile Chaix, Philippe Gerber, Michel Desprès, Lise Gauvin, Olivier Klein, Sylvain Klein, Bernhard Köppen, Sébastien Lord, Alexandre Naud, Marion Patte, Hélène Payette, Lucie Richard, Pierre Rondier, Martine Shareck, Cédric Sueur, Benoit Thierry, Julie Vallée, Rania Wasfi

**Affiliations:** Montreal University Research Center (CRCHUM), 850, rue St-Denis, Montréal, QC H2X 0A9 Canada; École de Santé Publique de l’Université de Montréal (ESPUM), 7101, rue du Parc, Montréal, QC H3N 1X9 Canada; Inserm, UMR-S 1136, Pierre Louis Institute of Epidemiology and Public Health, Faculté de médecine Saint-Antoine, 27 rue Chaligny, cedex 12, Paris, 75571 France; Luxembourg Institute of Socio-Economic Research, 11, Porte des Sciences, Esch-sur-Alzette, L-4366 Luxembourg; École d’urbanisme et d’architecture de paysage, Université de Montréal, 2940, chemin de la Côte-Sainte-Catherine, Montréal, QC H3C 3J7 Canada; UMR Géographie-Cités, 13 rue du Four, Paris, 75006 France; Canada Research Center on Aging, CIUSSS de l’Estrie-CHUS, Faculty of Medicine & Health Sciences, University of Sherbrooke, Sherbrooke, QC Canada; Faculty of Nursing, Université de Montréal, Montreal, QC H3C 3J7 Canada; London School of Hygiene and Tropical Medicine, London, UK; Département d’Ecologie, Physiologie et Ethologie; Institut Pluridisciplinaire Hubert Curien, 23, rue Becquerel, Strasbourg, 67087 France

## Erratum

After publication of this work [1], we noted that we inadvertently failed to include the complete list of all coauthors: co-author Marion Patte was missed. In addition, the affiliations for Cédric Sueur and Julie Vallée were mistakably reversed. The full list of authors, corrected author affiliations and the updated Authors Contributions section are reported here, and have also been corrected in the original article. We apologize for any inconvenience this oversight may have caused.

### Authors’ contributions

YK, BC and PG are the principal investigators of the CURHA study. Together with LG, SL, HP, MP, LR, OK, CS, BT, AN, JV, RW they conceived and designed the study. BK, MS and PR have/are coordinating the data collection campaigns in Canada and Luxembourg. All authors revised and approved the submitted manuscript.
